# Genetics of Lactose Intolerance: An Updated Review and Online Interactive World Maps of Phenotype and Genotype Frequencies

**DOI:** 10.3390/nu12092689

**Published:** 2020-09-03

**Authors:** Augusto Anguita-Ruiz, Concepción M. Aguilera, Ángel Gil

**Affiliations:** 1 Department of Biochemistry and Molecular Biology II, Institute of Nutrition and Food Technology “José Mataix”, Center of Biomedical Research, University of Granada, Avda. del Conocimiento s/n. Armilla, 18016 Granada, Spain; augustoanguita@ugr.es (A.A.-R.); caguiler@ugr.es (C.M.A.); 2 Instituto de Investigación Biosanitaria ibs.GRANADA, 18014 Granada, Spain; 3 CIBEROBN (Physiopathology of Obesity and Nutrition Network CB12/03/30038), Institute of Health Carlos III (ISCIII), 28029 Madrid, Spain

**Keywords:** epigenetics, genetics, lactase, lactase-phlorizin hydrolase, lactose intolerance, lactase persistence, lactase non-persistence

## Abstract

In humans the ability to digest milk lactose is conferred by a β-galactosidase enzyme called lactase-phlorizin hydrolase (LPH). While in some humans (approximately two-thirds of humankind) the levels of this enzyme decline drastically after the weaning phase (a trait known as lactase non-persistence (LNP)), some other individuals are capable of maintaining high levels of LPH lifelong (lactase persistence (LP)), thus being able to digest milk during adulthood. Both lactase phenotypes in humans present a complex genetic basis and have been widely investigated during the last decades. The distribution of lactase phenotypes and their associated single nucleotide polymorphisms (SNPs) across human populations has also been extensively studied, though not recently reviewed. All available information has always been presented in the form of static world maps or large dimension tables, so that it would benefit from the newly available visualization tools, such as interactive world maps. Taking all this into consideration, the aims of the present review were: (1) to gather and summarize all available information on LNP and LP genetic mechanisms and evolutionary adaptation theories, and (2) to create online interactive world maps, including all LP phenotype and genotype frequency data reported to date. As a result, we have created two online interactive resources, which constitute an upgrade over previously published static world maps, and allow users a personalized data exploration, while at the same time accessing complete reports by population or ethnicity.

## 1. Introduction

Lactose is the main carbohydrate present in milk and one of the main sources of energy during the nursing period in mammals. To take advantage of milk lactose, mammals have to first hydrolyze it into glucose and galactose, monosaccharides that can be easily absorbed by the intestinal tract [1]. In humans, the ability to digest milk lactose is conferred by a β-galactosidase enzyme called lactase-phlorizin hydrolase (LPH) [2,3]. The LPH enzyme is encoded by the lactase (LCT) gene, located on the chromosome 2q21. Exclusively expressed in the small intestine, in the apical part of microvilli within the brush border membrane of enterocytes, the LPH enzyme reaches the highest levels of activity during the nursing period [4]. After the weaning phase, however, in the majority of humans, the activity of LPH declines rapidly because of a decrease in the levels of the enzyme, and this trait is known as lactase non-persistence (LNP) [4,5,6]. As a consequence of LNP, the majority of humans are unable to digest lactose during adulthood, and some suffer clinical complications when they consume it. It has been estimated that approximately two-thirds of humans are LNP worldwide [7]. In the remaining third, however, there are individuals with the ability to digest milk and other lactose-rich dairy products during adulthood. This trait is known as lactase persistence (LP), and is particularly common in descendants from populations that have traditionally practiced cattle domestication [7,8]. Today, there is good functional evidence that the trait of LP has a genetic basis and follows a dominant pattern of inheritance [9]. Notably, it has been postulated that LP could be independently caused by, at least, five or more single nucleotide polymorphisms (SNPs) in a regulatory region (a transcriptional enhancer) called *MCM6* (minichromosome maintenance complex component 6)*,* which is located upstream of the LCT gene [9,10,11,12,13,14,15,16,17].

The frequency of LP is high in northern European populations, decreases across Southern Europe and the Middle East, and is low in non-pastoralist Asian and African communities [17,18,19,20]. Notably, LP is also common in pastoralist populations from Africa. It is hypothesized that natural selection has elicited a prime role in determining the current frequencies of LP in different human communities since the development of cattle domestication in the Middle East and North Africa around ~7500–9000 years before present (BP). Although LP and LNP frequencies have been widely studied worldwide, there is a lack of a work collecting all newly available information published to date [21,22,23,24,25,26,27,28,29,30,31,32]; especially the new frequency data reported for American populations. Given the great interest of this research topic, an online graphic resource gathering all reported lactase frequency data (both at the phenotypic and genetics levels) would be of much utility for the scientific community. With this purpose, interactive maps and other geographic information system (GIS) tools have been extensively employed with great success in other evolutionary biology areas [33]. Interactive mapping involves using maps that allow zooming in and out, panning around, identifying specific features, and querying underlying data, such as by topic or an accurate indicator (e.g., the allele frequency of a certain LP variant in a particular sub-population or ethnicity), generating reports, and other means of using or visualizing information in a map. This allows a more attractive way of presenting large sets of data that would not be sufficiently well exploited in static plots or large tables. Moreover, the online format of these resources allows easy access for researchers and constant update.

On the other hand, although great efforts have been devoted to elucidating the exact molecular mechanisms responsible for the natural decline in LPH activity after weaning (LNP), some gaps remain to be fulfilled [34]. Neither is there a unique, accepted theory about the evolutionary origin of LP and what was the advantage that it conferred to carriers, and that favored such a transmission across generations.

Taking all this into consideration, the aims of the present review were: (1) to gather and summarize all available information on LNP and LP genetic mechanisms and evolutionary adaptation theories, and (2) to create online interactive world maps, including all LP phenotype and genotype frequency data reported to date. As far as we are aware, this work constitutes the first interactive, and most updated, online resource for studying and exploring the phenotypic and genetic data on lactase phenotypes. This review is intended for general clinicians, nutritionists, dietitians, paediatricians, evolutionary biology researchers, geneticists, and any researcher working on the topic.

## 2. Materials and Methods

### 2.1. Systematic Review Methodology

The methodology to perform this systematic qualitative review encompasses the following processes, included in the preferred reporting items for systematic review and meta-analysis (PRISMA) statement [35,36]: definition of the research question, literature search, data collection, evaluation, comparison, and synthesis, as well as critical analysis and findings presentation, showing the strengths and weakness of the studies analyzed. A bibliographic search strategy was conducted to identify all studies focused on the genetic basis of lactase phenotypes in humans (LP and LNP). Furthermore, studies in animal and in vitro models were also considered, if they were purposed for investigating effects on the human *LCT* gene. The electronic databases consulted were PubMed, Scopus, Web of Science, and the Cochrane Central Register of Controlled Trials. The following medical subheading terms (MeSH) descriptors (“Lactase”, “Lactase-Phlorizin Hydrolase”, “Lactose Intolerance”, “Lactose Intolerance, Adult Type”) were used for the identification of records in PubMed, obtaining a total of 3822 articles (of which 516 were reviews). Next, a combination of all these MeSH terms along with the word “Genetics”, as a qualifier, derived a final depurated list of 40 articles. Those terms and additional non-MeSH terms (e.g., “hypolactasia”, “lactase persistence”, “lactase non-persistence”, and “lactose malabsorption”) were used for the identification of additional documents in the other databases. Additional records, not exclusively related to genetics but to other aspects of LP (such as population or ethnicity distributions, diagnosis or evolutionary biology), were also considered. Finally, works referenced in any selected record, and that were not present in performed Boolean searches, were also forced to inclusion, if they asserted inclusion criteria. This approach resulted in a total of 79 additional articles.

Inclusion criteria were papers written in English (with no geographical restrictions) published from 1 January 1960 to 20 July 2020, and the presence of the selected MeSH and non-MeSH terms in the title, abstract, or as keywords. Exclusion criteria were those studies focused on other forms of lactase deficiency different from the adult-type hypolactasia, such is the case of congenital or secondary hypolactasias. Articles studying the relation between lactase deficiency and other comorbidities (e.g., osteoporosis, cancer, diet disorders, etc.) were also excluded. The selection of manuscripts started by screening titles and abstracts for inclusion, creating a reference list of relevant papers. Two investigators (A.A.-R. and Á.G.) conducted each stage of document selection, deleted duplicate inputs, and reviewed studies as excluded or requiring further assessment. All data were extracted by one investigator (A.A.-R.) and cross-checked by a second investigator (Á.G.). In case of conflict, a third investigator (C.M.A.) took a final decision. A total of 103 studies were evaluated and finally selected after meeting the inclusion criteria, the application of the exclusion criteria, and an eligibility assessment. More details of the literature review are available in PROSPERO (https://www.crd.york.ac.uk/prospero/display_record.php?ID=CRD42020201258), and a flow chart of the different steps is available in the Supplementary Figure S1 as a PRISMA flow diagram [35,36].

### 2.2. Construction of Interactive World Maps

The whole data visualization process was implemented in R environment (Version 4.0.2) using the R package leaflet, one of the most popular open-source JavaScript libraries for interactive maps. The source code for the created visualization tool and the constructed input databases have been shared online, so they are available for update or extension to any other application. The software has been distributed as open-source software under the terms of the GNU Public License GPLv3, and it is hosted in the public hosting repository GitHub (https://github.com/AugustoAnguita/LactasePersistence_Interactive_WorldMaps).

## 3. Results and Discussion

### 3.1. Lactose: A Unique Sugar

The main carbohydrate in milk is the disaccharide lactose (β-d-Galactopyranosyl-(1→4)-d-glucose), which requires hydrolysis in the intestinal tract so that its component monosaccharides, galactose and glucose, can be taken up into the enterocytes and used later as a source of energy or structural elements. Lactose is found exclusively in mammal’s milk, although in a variable concentration, depending on the species. While humans have a mean composition of about 7 g of lactose per 100 mL of milk, other mammals present lower concentrations (e.g., 4.8, 4.1, and 4.8 g per 100 mL in cow, goat, and sheep milk, respectively) [37].

The hydrolysis of milk lactose in the intestine is catalyzed by the enzyme lactase-phlorizin hydrolase (LPH) (EC 3.2.1.108–EC 3.2.1.62), a β-galactosidase located in the brush border membrane of small-intestinal enterocytes. In humans, LPH is expressed only in the small intestine and is confined to absorptive enterocytes on the villi and not the proliferating cells of the crypts. Within the small intestine, LPH further demonstrates positional regulation, as exhibited by a tightly controlled pattern of expression along the proximal to the distal axis in both animals and humans, with high levels in the mid-intestine (mid-jejunum), and reduced levels in the duodenum and distal ileum [38,39,40]. This pattern of expression closely parallels that of another digestive hydrolase, i.e., sucrase-isomaltase [41]. However, lactase is expressed only at low levels throughout fetal life, whereas sucrase-isomaltase is expressed at high levels in the small intestine of early fetuses, and also transiently in the fetal colon [42].

### 3.2. The Lactase-Phlorizin Hydrolase Enzyme

In humans, the LPH enzyme is a 160 kDa transmembrane glycoprotein with a C-terminus (of 26 amino acids), which is intracellular, and an N-terminus, found at the luminal surface of the lipid bilayer of the microvillus membrane of enterocytes [1,3]. The transmembrane-spanning region is a short sequence of hydrophobic amino acids (19 residues). LPH is a multifunctional enzyme with several substrate specificities. Therefore, it can hydrolyze, in addition to lactose, lactosylceramide, cellobiose, cellotriose, flavonoid glucosides, and phlorizin. The primary sequence of the LPH protein in humans, rabbits, and rats reveals a four-fold internal homology (designated domains I–IV), most likely due to two independent duplication events during evolution [43]. It has been shown that domains I and II together serve to regulate protein folding in the endoplasmic reticulum; they are not glycosylated and they lack enzymatic activity. Domains III and IV are heavily glycosylated, are inserted into the microvillus membrane, and contain the two active sites in which each nucleophile is a glutamate residue. The amino acid sequence around the active site glutamic acid (E) in domain III is PIYITENG, whereas that of domain IV is PIYVTENG. First, nascent LPH (195 kDa) is synthesized in the endoplasmic reticulum and undergoes cotranslational, dolichol-dependent, high-mannose glycosylation, yielding a molecular mass of 215 kDa. Complex glycosylation of domains III and IV occurs in the Golgi, generating a structure of 220 kDa (O-glycosylations in serines and threonines as well as N-glycosylations in asparagine), and this glycosylation probably affects enzymatic activity as well as folding and intracellular transport [44]. Subsequently, there is a cleavage of a small N-terminal pro-enzyme, and of domains I and II that serve as a chaperone for the remaining molecule, which is inserted into the microvillus membrane. Finally, an extracellular cleavage event (perhaps carried out by pancreatic proteases) removes a small residue from the N-terminus, producing the final, mature LPH enzyme [45]. The role of the extra-cellular cleavage product remains unknown [3].

**Figure 1:**
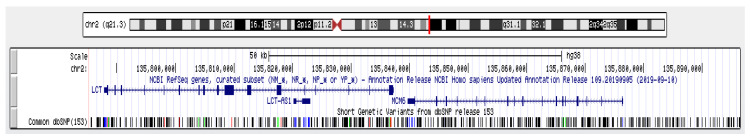
Genomic region 2q21 containing the *LCT* and *MCM6* loci (capture from the University of California, Santa Cruz (UCSC) Genome Browser). Exons are represented with boxes and introns with lines. This capture also includes information for all the known single nucleotide polymorphisms (SNPs) mapping the region, according to the dbSNP v153.

The human *LCT* gene, located on chromosome 2q21, comprises 17 exons and covers approximately 49 kb, giving rise to a messenger RNA of slightly more than 6 kb (Figure 1). From the initiation codon to the stop codon, the human *LCT* mRNA encodes 1927 amino acids, forming the complete translation product. Initial analyses of the gene identified several genetic variants within both the coding region and the 5′ -flanking region [43], although none were considered to have functional significance. Studies in transgenic mice have indicated that approximately 1 kb of the 5′ -flanking sequence of *LCT* in the pig, and 2 kb in the rat, are sufficient to direct appropriate tissue, cell, and villus expression, as well as a developmental decline after weaning [46,47,48]. Therefore, the majority of *LCT* regulatory elements may be located within, or surrounding, these regions. The first 100 bp of the proximal *LCT* promoter of the mammals analyzed to date (rat, mouse, pig, and human) are virtually identical, and appear to be similarly regulated. Interestingly, binding sites for caudal type homeobox 2 (Cdx-2), hepatocyte nuclear factor 1α (HNF1-α), and GATA transcription factors are in similar positions relative to the transcriptional start site [49,50,51,52]. In addition, a number of other transcription factors have been found to interact with the *LCT* 5′ -flanking sequence, albeit some in more distal loci, including HOXC11 (homeobox C11), HNF-3, C/EBP (CCAAT/enhancer binding protein), and FREAC-2/3 (forkhead box F2) [53,54,55,56], and have also been found to activate promoter–reporter constructs in transfection assays. In contrast, the transcription factor Pdx-1 (pancreatic and duodenal homeobox 1) appears to reduce the *LCT* expression in vitro [57]. As HNF1-α and GATA factors are found together in the intestine and colon, their interactions in the activation of *LCT* gene function have been extensively studied. In mouse intestine, the expression patterns of HNF1-α and GATA-4 closely correlate with *LCT* expression [51], being a physical interaction between these factors, necessary for the cooperative activation of the *LCT* promoter [52]. In contrast to the other mammals analyzed, the first 1000 bp of the 5′ -flanking region of the human *LCT* gene contains two Alu sequences of approximately 300 bp each, whereas the more distal region contains additional repetitive DNA sequences, making it difficult to directly compare this more distal regulatory region to those of other mammals. Whether inserted repetitive DNA segments affect *LCT* expression is still unknown. On the other hand, exon 17 of *MCM6*, a cell cycle regulatory gene ending 3.5 kb from the start site of the human *LCT* gene, seems to play a role as a regulatory enhancer of *LCT* [58]. The transcriptional start site of the *MCM6* gene lies approximately 39 kb 5′ of the *LCT* transcriptional start site. These two genes are close together, but the available evidence indicates that their regulation is independent [58].

### 3.3. Lactase Phenotypes and Their Genetic Origin

Lactase enzyme activity is high and vital during infancy, when milk is the main food. In most mammals, however, lactase activity declines after the weaning phase (to <10% of the neonatal values); with reduced activity maintained throughout adult life, and this is also the case in the majority of humans throughout the world who are described as LNP. This down-regulation is genetically programmed and seems to affect 70% of the world adult population. In other healthy humans (approximately a third of all humans) [59], especially people of Northern European ascendance and a few other population groups around the world, lactase activity persists at high levels throughout adult life, and this trait is known as LP. People who are LP can usually hydrolyze large amounts of lactose during adulthood and can thus consume large quantities of fresh milk without complication. People with LNP (also referred to as adult-type hypolactasia or lactase restriction) have a much lower lactose digestion capacity (LDC) than those with LP, and thus often, but not always, show symptoms after consumption of fresh milk [4]. When referring to LNP it is important to differentiate the term from others such as congenital lactase deficiency and secondary hypolactasia. Congenital lactase deficiency is extremely rare and appears at the onset of nursing, caused by severe mutations on both alleles of the *LCT* gene [60]. Secondary hypolactasia otherwise is caused by direct damage to mucosal lactase (e.g., celiac, giardiasis, enteritis), and does not have a genetic basis [61]. In this review, we will focus exclusively on the LNP and LP phenotypes, leaving behind any other type of hypolactasia. Other common terms usually employed concerning lactase phenotypes are lactose malabsorption and lactose intolerance, both referring to the consequences derived from an LNP status [62]. Lactose malabsorption refers to lowered blood glucose rise or increased breath hydrogen rise after an oral lactose load, and lactose intolerance describes the symptoms that normally accompany lactose malabsorption (e.g., diarrhea, farting, stomach pain, etc.).

**Figure 2:**
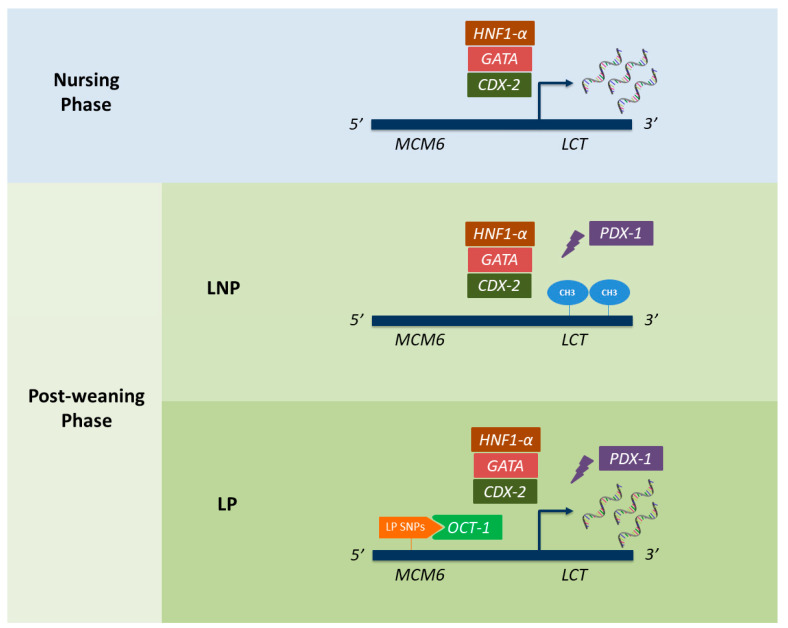
Genetic mechanisms underlying lactose persistence (LP) and lactase non-persistence (LNP) in humans. Rectangular shapes represent all the transcription factors currently known to interact with the lactase gene (LCT) promoter. While CDX-2, HNF1-α, GATA, and OCT-1 are known to promote LCT expression, the PDX-1 has been described as a transcriptional repressor. Oval shapes (named as “CH3”) refer to the appearance of methylations within the LCT region, which have also been described as repressing LCT expression. Finally, the different LP-associated alleles described in MCM6, and responsible for binding to OCT-1, are represented in orange. CDX-2: caudal type homeobox 2; HNF1-α: hepatocyte nuclear factor 1α; OCT-1: octamer-binding protein 1; PDX-1: pancreatic and duodenal homeobox 1.

About the genetics underlying LNP and LP, although considerable research efforts have been devoted, to date, some gaps remain unfulfilled. Concerning LNP, it seems that it is the direct action of transcriptional repressors, around the age of 5 years, which elicits the natural decline in lactase levels [46,47,48]. According to promoter studies, one plausible candidate could be the transcription factor Pdx-1, which has shown to directly downregulate *LCT* expression in vitro [57]. The reduction in the rate of LPH transcription would then elicit a consequent decrease in LPH-protein synthesis and enzyme activity along the proximal to the distal gradient, as well as the virtual extinction of expression in the duodenum and distal ileum. Besides the action of transcription factors, some epigenetics mechanisms (mainly DNA methylation) could also be involved in LNP regulation, as demonstrated by Labrie et al. (2016) [63]. Likewise, there have been described interpersonal differences in terminal glycosylation of the carbohydrate side chains of lactase, due to polymorphisms in Lewis (FUT3) and Secretor (FUT2) genes, corresponding to secretion of Lewis ABO (H) histo-blood group antigen CA19-9 [64]. Since these variants exert a direct influence on protein stability [44], not only DNA-binding elements, but also post-transcriptional factors, might contribute to the natural decline of intestinal lactase levels [5]. On the other hand, the persistence of LPH activity throughout adulthood (LP) seems to be a genetic trait following a dominant inheritance pattern, and involving the action of *cis*-element mutations. Particularly, it has been described that the effect of several SNPs leads to an alternative path for *LCT* expression that is not downregulated, as the original pathway is [4]. The molecular mechanism for these SNPs is the creation of new binding sites for specific transcription factors, such as the octamer-binding protein 1 (Oct-1) [65]. Interestingly, identified causal mutations seem not to be at *LCT* itself but 14 kb chromosomally upstream of *LCT,* on the same haplotype, in an intron of the previously introduced *MCM6* locus. As we have already mentioned, the *MCM6* has been described as a regulatory enhancer that modulates *LCT* expression, and hence protein levels [4,16,66]. A graphical summary of all the plausible genetics mechanisms involved in the development of lactase phenotypes can be found in Figure 2.

The identification of lactase phenotypes in humans (LP and LNP) can be done directly by assay of the lactase activity from a small intestine biopsy, or indirectly by lactose-tolerance tests [67]. Although the direct determination of lactase activity in intestinal samples is always better than lactose-tolerance tests, it constitutes an invasive and impractical technique for all situations. Among alternative lactose-tolerance tests, it highlights the measurement of an increase in blood glucose after giving a lactose load of 50 g, so that people with high lactase activity will present a significant rise in blood glucose concentration within 15 to 45 min, following lactose administration. Another lactose-tolerance test, and preferable over the blood glucose test, involves testing breath hydrogen after a lactose load or, ideally, after the ingestion of one serving (250 mL) of pasteurized or sterilized milk [68]. In LNP people, undigested lactose reaches the colon, where it is fermented, leading to the production of fatty acids and gases, including hydrogen, which is excreted in the breath. According to the genetic basis of LP, several genetic tests have also become available as alternatives to lactose tolerance tests and intestinal biopsies. Although these genetic tests constitute non-invasive and useful diagnostic tools, depending on the genetic variants analyzed, they are not always appropriate for all populations.

### 3.4. Lactase Phenotypes and Their Genetics Origin

**Figure 3:**
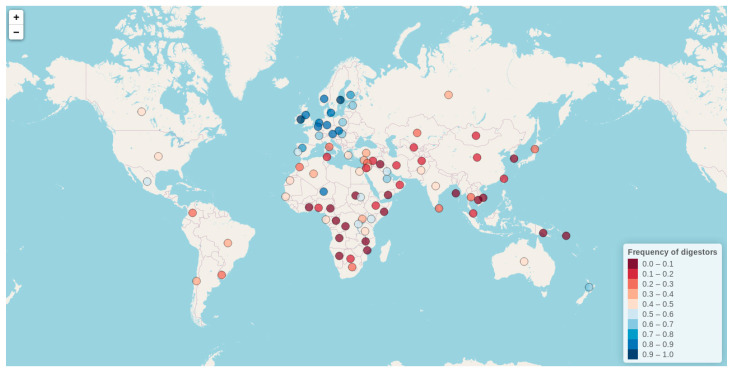
Interactive map exploring the lactase persistence (LP) frequencies worldwide (http://bionit.ugr.es/pages/investigacion/software/bioinformatics-methods-software). It includes all available literature reports on LP frequencies till 20 July 2020. It also offers a detailed description for each included study (incorporating information related to geographical location, ethnicity, number of individuals analyzed, reported frequency, and literature references). The figure presented here represents a static capture of the map (showing mean LP frequencies per country).

By means of either of these diagnostic tools, an impressive number of studies since the 1960s have investigated the distribution of lactase phenotypes worldwide, reporting sharply different LP frequencies among distinct human populations, and raising a provocative question in relation to evolutionary genetics. With their reviews, Itan et al. (2010) and Storhaug et al. (2017) gathered all available LP frequency phenotype data until 2016, becoming a reference for the study of LP phenotype worldwide distributions. Nonetheless, no review study has been recently published with updated data. Taking this into consideration, in the present work, we have collected all LP frequency data available till 2020 (also including the data from Itan et al. (2010), and Storhaug et al. (2017)) (Supplementary Table S1), and generated an interactive world map. Our interactive map constitutes the most comprehensive and updated resource for exploring LP frequencies worldwide. Besides including all available literature reports on LP frequencies till 20 July 2020, our map further offers a detailed description for each included study (incorporating information related to geographical location, ethnicity, number of individuals analyzed, reported frequency, and literature references). One of the greatest virtues of our interactive map is its ability to organize large amounts of data in a single shot, which otherwise would be hosted in unpleasantly big tables. As a fully extendable and interactive graphic, our tool further constitutes an upgrade over previously published static world maps, allowing users a personalized data exploration at the same time as accessing complete reports by population. For the construction of our database and interactive map, LP frequency data obtained either by intestinal biopsy or any type of presented lactose-tolerance and genetic tests have been considered. Although the interactive map is hosted at (http://bionit.ugr.es/pages/investigacion/software/bioinformatics-methods-software), in the present article we show a static capture of the map (showing mean LP frequency values per country) (Figure 3).

As can be observed, the frequency of LP varies greatly among populations, ranging from 0% to almost 100%, with the highest rates found in people of northern European descent and some populations from West Africa, East Africa, and the Middle East (http://bionit.ugr.es/pages/investigacion/software/bioinformatics-methods-software). The frequency decreases as one moves to the south and east of the map (with 30% of LP reported in Italy). This descending gradient is disrupted in some European countries with a strong cultural admixture tradition, such is the case of Spain (with reported frequencies ranging from 47% to 91%). Interestingly, LP is particularly frequent in some milk consuming nomads and pastoralist communities of the Afro-Arabian area in comparison to their neighboring populations (e.g., 86% in Beduin Saudi, 88% in Ben-Amir, 80% in Haddendoa, and 70% in Fulani). There is a very low level of LP in the Asiatic countries (15% in China, and 0% in South Korea, Vietnam, and Cambodia). Regarding the USA and Mexico, although they present a relatively low mean LP frequency (48 and 52% respectively), highly variable LP sub-frequencies, according to ethnicity, have also been reported. For example, while LP is present in 83% to 93% of white Americans, with European or Scandinavian extraction, it shows a frequency of around 30% in Mexicans from rural areas, and 12–40% in African Americans. Conversely, LNP is found in 83% of Ashkenazi Jews in Israel, and in 81–92% of pure-bred American Indians. Low LP frequencies have also been found generally in South America (with 20% in Colombia, 6% in Peru, 38% in Chile, 37% in Brazil, and 30% in Uruguay), except for some white-ancestry Mennonite sub-populations in Brazil.

Although LP has been investigated in an impressive number of individuals, some geographical areas remain phenotypically understudied, notably in North Africa (e.g., Tunisia, Libya, Mali, or Mauritania), West Africa, and specific locations around the Caucasus. The situation should also be clarified in the many Caribbean and South American countries, such as Argentina, Bolivia, Ecuador, Uruguay, Venezuela, Dominican Republic, Panamá, Guatemala, or Cuba, for whom we lack LP phenotype data entirely.

### 3.5. Current Status of Genetics Aetiology of Lactase Persistence

The genetic basis for population variation in lactase production as a dominant trait is well-described, although not yet complete, with *cis*-element mutations responsible for LP identified in the transcriptional enhancer *MCM6* [9,10,11,12,13,14,15,16,17]. Although LP has been known for almost a century, it was not until 2002 that the first LP mutation was discovered [69]. Such a delayed discovery was probably caused by the location of the LP alleles, which map 14 kb upstream of the *LCT* gene and not within, or immediately upstream, of it. Among identified variants, the −13910:C>T (rs4988235) [69] has almost reached fixation in some parts of Europe, while others such as −13907:C>G (rs41525747), −13915:T>G (rs41380347), −14009:T>G (rs869051967), and −14010:G>C (rs145946881) are found at variable frequencies in the Middle East and Africa [10,12,14]. Besides being highlighted as the most widespread and strongly associated LP variants, these five SNPs have been reported as functional markers according to a vast range, of both in vitro transfection assays, and in vivo studies [10,12,13,16,39,65,66,70,71,72,73,74].

In addition to these five genetic markers, up till now, eighteen new SNPs mapping the *MCM6* have also been associated with LP in specific populations, thereby making a total of twenty-three known SNPs that currently underlie the genetic etiology of LP (Table 1). Interestingly, these variants seem to have arisen during the same time period, but independently in different human populations, and this is the reason why LP has become a textbook example of convergent regulatory evolution, and gene-culture co-evolution. In the presented Table 1, we display the known identifiers for mentioned variants as well as any evidence of functional control on *LCT* expression according to the literature. Analysis of an 80 kb haplotype covering the region of *LCT* and the upstream *MCM6* enhancer has further confirmed a tight association of these LP variants with particular haplotypes [10,12,14], and shows that haplotype diversity also differs between populations, with the least diversity observed in Northern Europeans [9].

**Figure 4:**
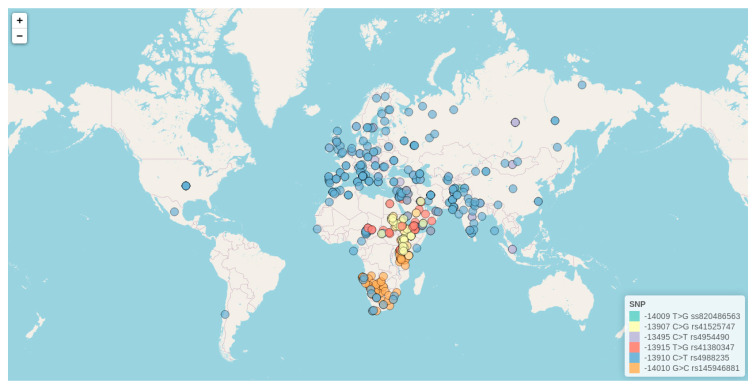
Interactive map exploring worldwide allele frequencies for the most studied lactase persistence (LP)-associated genetic variants (http://bionit.ugr.es/pages/investigacion/software/bioinformatics-methods-software). Data for each SNP are represented by a different color. It also offers a detailed description for each included study (incorporating information related to geographical location, ethnicity, frequency of LP-associated alleles, and literature references). The figure presented here represents a static capture of the interactive map.

Of the twenty-three genetic variants included in Table 1, there are ten with a rare frequency (q < 5%) in the populations where they were discovered, as well as another SNP in complete linkage disequilibrium (LD) with the well-known −13.910:C>T. For the remaining twelve frequent and widely studied SNPs, we have created a database including all available information for minor allele worldwide frequencies reported till July 2020 in the literature (Supplementary Table S2). For the most genotyped variants among these twelve markers (those SNPs that have been studied in more than ten different populations around the world), we have further created an interactive map following the same format as the one previously presented for LP phenotype data. The six genetic variants represented in the interactive map comprise −13910:C>T (rs4988235), −13495:C>T (rs4954490), −14009:T>G (rs869051967), −13907:C>G (rs41525747), −13915:T>G (rs41380347), and −14010:G>C (rs145946881)), for which we further include information related to ethnicity, number of genotyped individuals, reported minor allele frequencies, and literature references. This interactive map is hosted at (http://bionit.ugr.es/pages/investigacion/software/bioinformatics-methods-software) and a static capture is presented in Figure 4. For the construction of such a database and interactive map, genotype data available in the work from Liebert et al. (2017) [9] as well as in any additional genetic study published from 2016 to 2020 have been considered. Owing to that, our map constitutes the first resource presenting distribution data for LP associated alleles in American populations.

As can be observed in the map, highly variable SNP frequencies have been described worldwide, differing not only between countries but also between different sub-populations or ethnicities within the same geographical location. By far, the −13.910:C>T (rs4988235) is the more widely extended and studied variant, with a presence in lots of ethnicities and cultures. First identified in 2002, in a study of Finnish families by Enattah et al. (2002) [69], this variant has been further confirmed as a *cis*-acting enhancer of the *LCT* promoter in both in vitro and in vivo studies [13,66,75]. The molecular mechanism through which the T allele of this variant increases LCT expression consists of the creation of a new binding site for the Oct-1, a transcription factor that interacts with human HNF1-α to bind to the *LCT* promoter [65]. Therefore, this allele leads to an alternative path for *LCT* expression that is not downregulated, as the original path is. Interestingly, in vivo studies further revealed that, even though the LP phenotype is considered a binary trait encoded dominantly, lactase activity is instead a codominant quantitative trait, with a clear trimodal distribution [76]. This difference in expression between homozygous and heterozygous likely has a minor effect on the ability to efficiently break down lactose, but as suggested by Swallow, it could become important under certain conditions, such as stress or disease. Although Enattah et al. (2002) [69] also reported another mutation associated with the LP phenotype, the −22.018:G>A (rs182549), it has been demonstrated that it is in complete LD with −13.910:C>T, and not able to drive LP by itself. Actually, −13.910 and −22.018 might interact epistatically; in vitro studies have shown that the −22.018 region is a weak silencer of the enhancer activity driven by −13.910 [16,39,66]. As can be observed in our map, −13.910:C>T is not only a European mutation but also underlies the LP phenotype all over Asia, including in various populations from Russia, Pakistan, and Iran; Central Asia (prevalence of 30% in herders); and Nepal (prevalence of up to 34% in herders from the north) [77,78]. It is also the primary LP-associated mutation in Algerians (17–33%), Mozabites (22%), and other Berber populations (14–23%) from North Africa, as well as in Fulani (23–48%) from both Central Africa (Cameroon) and East Africa (South Sudan). Interestingly, the −13.910:C>T has also been found at a moderate frequency in South America (22% in communities from the Coquimbo region in Chile) [25,79], Central America (20% in rural areas from Mexico), and in North America (21–82%).

Although it was thought that in all of Eurasia the LP phenotype was monogenic, a second variant, the −13495:C>T, was described and found to be widely spread worldwide (87% prevalence in Norway, 96% in Ireland, 78% in UK, 50% in Spain, Italy, and Portugal, as well as a frequency higher than 15% in other Eurasians regions such as Azerbaijan, Georgia, Uzbekistan, Russia, Mongolia, Pakistan, and India). In all the populations where the −13495:C>T has been found, the variant exhibits very similar frequencies to the −13.910:C>T, with a practically complete LD described for both markers (D’ = 0.99 according to the 1000 Genomes Project Phase 3). Located just outside the enhancer *MCM6* region, the −13495:C>T has been shown to occur as a derived allele on an ancestral haplotype and to be older than the −13.910:C>T [9].

The situation is different in the Arabian peninsula and East Africa, where four different mutations have been found to be associated with LP: −13.907:C>G (rs41525747), −13.915:T>G (rs41380347), −14.009:T>G (ss820486563), and −14.010:G>C (rs145946881), all of which cluster in the intron 13 of the *MCM6*, within a 100-base-pair interval of each other that includes the −13.910:C>T [12,14,16,65,66,74]. All of these mutations result in an increase of lactase activity in vitro [10,12,13,16,39,65,66,70,71,72,73,74], and two of them, −13.907:C>G and −13915:T>G, seem to affect binding of Oct-1 [10,73]. While −14.010:G>C is most prevalent as one goes to East Africa (32–46%) [12] and South Africa (13–20%) [80,81], −13.907:C>G and −14.009:T>G are most prevalent among the Beja people of Sudan and in Ethiopia. On the other hand, −13915:T>G is the most common variant in camel herders from the Middle East (72–88%) [10,82], and is also extended among almost all Central-East African populations (Kenya, Sudan, Cameroon, Nigeria, Chad).

Although increasing data are becoming available for American populations, these have been exclusively focused on the −13.910:C>T variant. Given the great diversity of Afro-descendants living in the American continent, more studies focused on the new variants identified by Tishkoff et al. (2007) [12] (−13.907:C>G (rs41525747), −13.915:T>G (rs41380347), −14.009:T>G (ss820486563), and −14.010:G>C (rs145946881)) would be of much interest, and would complete the picture for worldwide LP alleles distributions.

### 3.6. Evolutionary Genetics of Lactase Persistence

All LP alleles show strong signals of positive selection [83] and have spread through migration [84]. Their evolutionary advantage has been argued in the ability they confer to humans to consume animal milk during adulthood without risk of symptoms of lactose intolerance [12,84,85,86,87,88,89]. Besides a positive selection mechanism, present-day frequencies of these alleles have also been clearly influenced by other processes, including population expansion, allele surfing, and cultural/environmental processes [84]. According to ancient DNA studies, the earliest occurrences of an LP allele (the −13.910:C>T) have been reported in Spain, dated to about 5000 years BP according to PCR data [90], or in Germany and Sweden about 4000 BP according to NGS data [85,88,91,92]. This is in tune with performed extended haplotype homozygosity (EHH) analyses of the sequences surrounding LP SNPs, which have shown that the haplotypes carrying the derived LP-associated alleles are much longer than their ancestral counterparts, supporting a recent origin of these variants [93]. Accordingly, by means of other population genetic and modeling approaches, some authors have estimated the strength and timing of selection from a sample of European Americans, and found that the −13.910:C>T allele arose 2188–20,650 years BP, and that this selection was favored with a selection coefficient of 0.014–0.15 [10,86]. Such selection coefficients place the LP as one of the strongest examples of positive selection in the human genome, even ahead of the resistance to malaria (0.04–0.09) [94], and skin pigmentation in Europeans (0.03) [95].

Four main theories of evolutionary advantage have been proposed to explain the observed strong LP selection coefficients and the high LP frequencies found in the majority of dairying populations worldwide [reviewed in [7,96,97]:

- Lifelong access to nutrient-rich milk;
- Lifelong access to carbohydrate and fluid source, critical to pastoralists living in hot, arid environments;
- Lactose enhances calcium absorption, which may be compromised by low vitamin D synthesis in high latitude (northern) environments;
- Human consumption of bovine milk may accelerate reproductive maturation or physical growth, or contribute to larger adult size, possibly due to milk’s stimulatory effects on insulin-like growth factor-I.

For non-dairying groups, however, the above hypotheses suggest that LP mutations did not spread, as they did not confer any advantage.

Although these four theories are widely accepted and serve as the best explanation for the majority of scenarios, there remain some questions regarding the evolutionary origin of LP. This is particularly the case with some herders from the Middle East, where low LP frequencies have been found even though they have relied heavily on pastoralism for millennia (12% and 24% in long-term Mongol and Kazakh herders, respectively) (http://bionit.ugr.es/pages/investigacion/software/bioinformatics-methods-software) [97]. Central Asian herders are not the only exception to the expected pattern. Indeed, the Sami reindeer herders in Scandinavia have a lower LP frequency than the rest of the Swedish population (33% versus 91%) despite a higher dependence on pastoralism (60% versus 30%). Similarly, some African pastoral ethnic groups who consume milk (50–90% pastoralism) have low LP frequencies, as in the case of the Dinka (LP frequency of 0–25%) and Nuer (22%) in Sudan, the Somali in Ethiopia (24%), and the Herero in South Africa (3%). Furthermore, in the areas where animals were first domesticated (notably in Turkey) and more generally around the Mediterranean, populations that have used milk for millennia have moderate LP frequencies.

Two main hypotheses have been postulated to explain such discrepancies: (1) The existence of patterns of admixture between pastoral and non-pastoral populations, which could have limited the efficacy of natural selection, and (2) the ability to transform milk into derived dairy products, which would has been further affected by a combination of cultural, nutritional, and environmental issues (such as the preference or need to ferment milk, the stability and availability of other sources of energy, seasonality and mobility limitations or the type of livestock). The admixture between pastoral and non-pastoral populations could explain the situation found in Central Asia, which is a migratory crossroads, and is located in the middle of non-pastoral groups. Otherwise, the ability to ferment milk might account for the intermediate LP frequencies found in populations around the Mediterranean and north of the Middle East (39%), where dairy animals were first domesticated, given that these populations consume moderate amounts of fresh milk (102 L per person per year) and transform a large proportion of milk into cheese and fermented milk (38% on average) [98]. On this matter, it has been demonstrated that variability in fermenting practices correlates better with LP frequency than the levels of pastoralism [97].

### 3.7. New Insights into the Genetic Basis of Lactase Phenotypes: Epigenetics Alterations

Despite the identification of LP-associated alleles, some additional molecular basis of the trait remains to be uncovered. Indeed, two populations with substantial LP have been reported to completely lack known LP-associated mutations: the Hadza from Tanzania, who have an LP frequency of 47% [12,99], and the Wolof from Senegal, who have an LP frequency of 51% [74,100]. The case of the Hadza is particularly intriguing because no associated mutation has been found despite a large sequencing effort, including intron 9 of *MCM6* (1.3 kb), intron 13 of *MCM6* (3.2 kb), and the *LCT* promoter (2 kb) [12,99]. As complementary underlying mechanisms, some authors have postulated that lactase phenotypes could also be directed by epigenetic modifications [31,63,101,102,103]. The first evidence supporting this hypothesis was published by Labrie et al. (2016) [63], who demonstrated that the C allele from the −13910:C>T was associated with the appearance and accumulation of age-related methylations at both the *MCM6* and the *LCT* regions [63]. Indeed, they demonstrated that these DNA variation-dependent methylations further elicit a silencing on *LCT* expression, thereby being a plausible contributor to LNP in mammals. In line with Labrie’s findings, another work demonstrated how certain epigenetic signatures, at enhancer and promoter *LCT* elements, align with transcriptional variation of *LCT* in mouse enterocytes [103], further reporting how age and phenotype-specific environmental cues (lactose exposure after weaning) induce changes to these epigenetics modifications and alter the intestinal *LCT* mRNA gradient [103]. Finally, a recent study identified putative *LCT* meQTLs (methylation quantitative trait loci), which are differentially methylated between LP and LNP individuals [31]. Interestingly, these authors also found that DNA methylations in the enhancer and promoter sites of the *LCT*, rather than the expression of intestinal transcription factors such as CDX2, POU2F1, GATA4/6 or HNF1-α, were predictive of LNP/LP (Figure 2) [31]. Indeed, DNA methylation variation presented a higher power to predict lactase phenotypes than the classic LP alleles alone.

Altogether, results from these studies suggest a plausible role of epigenetics in the appearance and regulation of lactase phenotypes, and reinforce the utility of DNA methylation as a complementary biomarker for LP diagnosis. More studies would be needed in this area in order to completely elucidate the epigenetic etiology of the trait. If demonstrated, a worldwide distribution map for such epigenetic signatures would also be of much value for the scientific community.

### 3.8. Lactose Intolerance and Intestinal Microbiota

Another aspect of special relevance for the understanding of lactase phenotypes is the human microbiota, which has been described to elicit a direct influence on the appearance, or amelioration, of lactose intolerance clinical symptoms [104,105]. This relationship results from the ability of human colonic microbiota (the sum of all microorganisms present in the colon) and their genes (intestinal microbiome) to ferment undigested lactose. This fermentation ability varies widely among individuals, and depends mostly on the composition of each colonic microbiota. This is important to the point that we may observe an amelioration or a worsening of lactose intolerance symptoms according to the abundance of certain strains of bacteria in the colon of individuals. For example, if colonic bacteria are efficient at fermenting lactose, then the lactose osmotic shock (which leads to diarrhea) will be reduced, but the quantity of gases might increase. In parallel, if methanogenic archaebacteria are prevalent, then carbon dioxide will be transformed mostly into methane, leading to constipation rather than diarrhea, as observed in 30% of intolerant individuals [106]. In addition, consumption of dairy products by LNP individuals can influence the colonic microbiota composition and lead to a reduction of intestinal symptoms [107,108]. Based on the fact that intestinal microbiota can be modulated by supplementation, many studies have tried to induce a “healthy” microbiota in LNP subjects, in order to alleviate the signs and symptoms of lactose intolerance. As a result, a positive relationship has been found between probiotics and lactose intolerance, in relation to specific strains and concentrations [105]. Taking all this into consideration, diet-induced particularities in human microbiota could be an additional explanation for those discrepancies encountered in LP/LNP phenotypes and discussed in the present review.

## 4. Conclusions

Lactase phenotypes (LP and LNP) in humans present a complex genetic basis that has been widely investigated during the last decades. Although great advances have been achieved, the exact molecular mechanisms underlying the appearance of LNP after the weaning phase remain not fully understood. Probably, the combined action of transcription factors and epigenetics alterations on the *LCT* gene is the most plausible explanation. In the present review, we have summarized all proposed explanatory theories and gathered them in a figure (Figure 2). The mechanisms responsible for LP, on the other hand, are known in better detail, and respond to the existence of *cis*-element mutations mapping a gene other than *LCT*; the regulatory enhancer region *MCM6*. Particularly, there have been a total of twenty-three SNPs within the *MCM6* associated with LP so far in human populations. These variants seem to have arisen during the same period but independently in different human populations, the reason why LP has become a textbook example of convergent regulatory evolution and gene-culture co-evolution.

The distribution of lactase phenotypes, and their associated SNPs across human populations, has also been widely studied though not recently reviewed. All available information has always been presented in the form of static world maps or large-dimension tables, so that it would benefit from the newly available visualization tools, such as interactive world maps. In the present review, besides gathering all information available to date for LP phenotype and genetic frequencies, we have also created two online interactive resources (http://bionit.ugr.es/pages/investigacion/software/bioinformatics-methods-software). As fully expandable and interactive graphics, our tools further constitute an upgrade over previously published static world maps, allowing users a personalized data exploration at the same time as accessing complete reports by population or ethnicity. Despite the great availability of data, we have detected that more studies are needed, especially for certain Central and South America regions, for which LP phenotype/genetics data lack entirely. Likewise, there is also a need for more genetics studies on the frequency of genetic variants other than the −14010:G>C (especially in non-Caucasian populations). Finally, and due to the existence of certain studies pointing to epigenetics as a plausible contributor to lactase phenotypes, it would also be interesting to carry out more population studies focused on the distribution of *LCT* epigenetics signatures associated with LP/LNP.

Concerning evolutionary genetics, LP is one of the strongest examples of positive selection found in the human genome. According to the latest studies, the evolutionary advantage conferred by LP alleles mainly lies in the lifetime access to nutrient-rich milk in populations that have traditionally practiced cattle domestication. However, there are still some discrepancies unexplained, as is the case of several ancient pastoral populations with a very low or null frequency of LP. As possible explanations, variability in milk fermentation practices and population admixture events have been proposed. On this matter, more nutritional anthropological studies investigating the amount, type, and seasonality of milk and dairy products consumed, and the perception of these foods in traditional populations would be helpful in understanding why some populations took up drinking fresh milk, whereas others mostly transformed it.

Human gut microbiome is, in a way, an extension of the human genome; as thousands of metabolic processes performed by members of the microbial community directly influence host physiology, including their host’s ability to utilize lactose and other carbohydrates. Hence, further studies of utilization of lactose by the human microbiome are needed to explain discrepancies found in LP/NLP phenotypes.

Research works like the present review lay the foundations for the study of the genetic bases of lactase phenotypes in humans, and represent a new paradigm in the way of visualizing genetic and phenotype data at the population level.

## Figures and Tables

**Table 1:** Lactase persistence (LP)-associated genetic variants in the MCM6.

SNP	RS-id	Additional Information
−14010:G>C **	rs145946881	Widely studied and associated
−14009:T>G **	rs869051967	Widely studied and associated
−13915:T>G **	rs41380347	Widely studied and associated
−13910:C>T **	rs4988235	Widely studied and associated
−13907:C>G **	rs41525747	Widely studied and associated
−22.018:G>A	rs182549	In complete LD with the causal −14010:G>C
−14011:C>T *	rs4988233	
−13906:T>A		
−13779:G>C *	rs527991977	
−13744:C>G		
−13730:T>G	rs4954492	
−13603:C>T	rs56348046	
−13495:C>T	rs4954490	
−13914:G>A		Rare variant (*q* < 5%)
−14062:G>A		Rare variant (*q* < 5%)
−14028:T>C	rs759157971	Rare variant (*q* < 5%)
−13753:C>T		Rare variant (*q* < 5%)
−13693:G>A		Rare variant (*q* < 5%)
−13806:A>G	ss820496565	Rare variant (*q* < 5%)
−13964:C>A		Rare variant (*q* < 5%)
−13771:A>G		Rare variant (*q* < 5%)
−14010 G>A	rs145946881	Rare variant (*q* < 5%)
−13926 A>C		Rare variant (*q* < 5%)

For all included genetic variants, we report any available identifier as well as any evidence of functional control on LCT expression according to the literature. Evidence of functional role in controlling LCT expression is indicated as follows: ** for validated functional SNPs, * some evidence for functional SNPs. Abbreviations: LD, Linkage Disequilibrium; RS-id, Reference SNP identifier; SNP, Single Nucleotide Polymorphism.

## Supplementary Materials

The following are available online at https://www.mdpi.com/2072-6643/12/9/2689/s1, Figure S1: PRISMA 2009 Flow Diagram for adopted Systematic Review Methodology, Table S1: Phenotypic data for lactase persistence (LP) frequencies worldwide (raw data for Figure 3). Table S2: Allele frequencies for the most studied lactase persistence (LP)-associated genetic variants (raw data for Figure 4).

## References

[1] Büller H.A., Grand R.J. (1990). Lactose Intolerance. Annu. Rev. Med..

[2] Montgomery R.K., Büller H.A., Rings E.H.H.M., Grand R.J. (1991). Lactose intolerance and the genetic regulation of intestinal lactase-phlorizin hydrolase. FASEB J..

[3] Naim H.Y. (2001). Molecular and cellular aspects and regulation of intestinal lactase-phlorizin hydrolase. Histol. Histopathol..

[4] Swallow D.M. (2003). Genetics of Lactase Persistence and Lactose Intolerance. Annu. Rev. Genet..

[5] Rossi M., Maiuri L., Fusco M., Salvati V., Fuccio A., Auricchio S., Mantei N., Zecca L., Gloor S., Semenza G. (1997). Lactase persistence versus decline in human adults: Multifactorial events are involved in down-regulation after weaning. Gastroenterology.

[6] Lee M.-F., Krasinski S.D. (2009). Human Adult-Onset Lactase Decline: An Update. Nutr. Rev..

[7] Ségurel L., Bon C. (2017). On the Evolution of Lactase Persistence in Humans. Annu. Rev. Genom. Hum. Genet..

[8] Weiss K.M. (2004). The unkindest cup. Lancet.

[9] Liebert A., López S., Jones B.L., Montalva N., Gerbault P., Lau W., Thomas M.G., Bradman N., Maniatis N., Swallow D.M. (2017). World-wide distributions of lactase persistence alleles and the complex effects of recombination and selection. Hum. Genet..

[10] Enattah N.S., Jensen T.G.K., Nielsen M., Lewinski R., Kuokkanen M., Rasinpera H., El-Shanti H., Seo J.K., Alifrangis M., Khalil I.F. (2008). Independent Introduction of Two Lactase-Persistence Alleles into Human Populations Reflects Different History of Adaptation to Milk Culture. Am. J. Hum. Genet..

[11] Gibson G. (2007). Human Evolution: Thrifty Genes and the Dairy Queen. Curr. Biol..

[12] Tishkoff S.A., Reed F.A., Ranciaro A., Voight B.F., Babbitt C.C., Silverman J.S., Powell K., Mortensen H.M., Hirbo J.B., Osman M. (2007). Convergent adaptation of human lactase persistence in Africa and Europe. Nat. Genet..

[13] Fang L., Ahn J.K., Wodziak D., Sibley E. (2012). The human lactase persistence-associated SNP −13910*T enables in vivo functional persistence of lactase promoter–reporter transgene expression. Hum. Genet..

[14] Ingram C.J.E., Elamin M.F., Mulcare C.A., Weale M.E., Tarekegn A., Raga T.O., Bekele E., Elamin F.M., Thomas M.G., Bradman N. (2007). A novel polymorphism associated with lactose tolerance in Africa: Multiple causes for lactase persistence?. Hum. Genet..

[15] Jensen T.G.K., Liebert A., Lewinsky R., Swallow D.M., Olsen J., Troelsen J.T. (2011). The −14010*C variant associated with lactase persistence is located between an Oct-1 and HNF1α binding site and increases lactase promoter activity. Hum. Genet..

[16] Troelsen J.T., Olsen J., Møller J., Sjöström H. (2003). An Upstream Polymorphism Associated with Lactase Persistence has Increased Enhancer Activity. Gastroenterology.

[17] Itan Y., Jones B.L., Ingram C.J., Swallow D.M., Thomas M.G. (2010). A worldwide correlation of lactase persistence phenotype and genotypes. BMC Evol. Biol..

[18] Storhaug C.L., Fosse S.K., Fadnes L.T. (2017). Country, regional, and global estimates for lactose malabsorption in adults: A systematic review and meta-analysis. Lancet Gastroenterol. Hepatol..

[19] Büning C., Schmidt H., Lochs H., Ockenga J. (2004). Genetic Components of Lactose Intolerance and Community Frequency. J. Bone Miner. Res..

[20] Bayless T.M., Brown E., Paige D.M. (2017). Lactase Non-persistence and Lactose Intolerance. Curr. Gastroenterol. Rep..

[21] Vicente M., Priehodová E., Diallo I., Podgorná E., Poloni E.S., Černý V., Schlebusch C.M. (2019). Population history and genetic adaptation of the Fulani nomads: Inferences from genome-wide data and the lactase persistence trait. BMC Genom..

[22] Priehodová E., Austerlitz F., Čížková M., Mokhtar M.G., Poloni E.S., Černý V. (2017). The historical spread of Arabian Pastoralists to the eastern African Sahel evidenced by the lactase persistence −13,915*G allele and mitochondrial DNA. Am. J. Hum. Biol..

[23] Zadro C., Dipresa S., Zorzetti G., Pediroda A., Menegoni F. (2017). Lactase non-persistent genotype distribution in Italy. Minerva Gastroenterol. Dietol..

[24] Hubácek J.A., Adámková V., Šedová L., Olišarová V., Adámek V., Tóthová V. (2017). Frequency of adult type-associated lactase persistence LCT-13910C/T genotypes in the Czech/Slav and Czech Roma/Gypsy populations. Genet. Mol. Biol..

[25] Montalva N., Adhikari K., Liebert A., Mendoza-Revilla J., Flores S.V., Mace R., Swallow D.M. (2019). Adaptation to milking agropastoralism in Chilean goat herders and nutritional benefit of lactase persistence. Ann. Hum. Genet..

[26] Brasen C.L., Frischknecht L., Ørnskov D., Andreasen L., Madsen J.S. (2017). Combination of real-time PCR and sequencing to detect multiple clinically relevant genetic variations in the lactase gene. Scand. J. Clin. Lab. Investig..

[27] Chin E.L., Huang L., Bouzid Y.Y., Kirschke C.P., Durbin-Johnson B., Baldiviez L.M., Bonnel E.L., Keim N.L., Korf I., Stephensen C.B. (2019). Association of Lactase Persistence Genotypes (rs4988235) and Ethnicity with Dairy Intake in a Healthy U.S. Population. Nutrients.

[28] Valencia L., Randazzo A., Engfeldt P., Olsson L.A., Chávez A., Buckland R.J., Nilsson T.K., Almon R. (2017). Identification of novel genetic variants in the mutational hotspot region 14 kb upstream of the LCT gene in a Mexican population. Scand. J. Clin. Lab. Investig..

[29] Bergholdt H.K.M., Nordestgaard B.G., Varbo A., Ellervik C. (2018). Lactase persistence, milk intake, and mortality in the Danish general population: A Mendelian randomization study. Eur. J. Epidemiol..

[30] Charati H., Peng M.-S., Chen W., Yang X.-Y., Jabbari Ori R., Aghajanpour-Mir M., Esmailizadeh A., Zhang Y.-P. (2019). The evolutionary genetics of lactase persistence in seven ethnic groups across the Iranian plateau. Hum. Genom..

[31] Leseva M.N., Grand R.J., Klett H., Boerries M., Busch H., Binder A.M., Michels K.B. (2018). Differences in DNA Methylation and Functional Expression in Lactase Persistent and Non-persistent Individuals. Sci. Rep..

[32] Ben Halima Y., Kefi R., Sazzini M., Giuliani C., De Fanti S., Nouali C., Nagara M., Mengozzi G., Elouej S., Abid A. (2017). Lactase persistence in Tunisia as a result of admixture with other Mediterranean populations. Genes Nutr..

[33] Kozak K.H., Graham C.H., Wiens J.J. (2008). Integrating GIS-based environmental data into evolutionary biology. Trends Ecol. Evol..

[34] Montgomery R.K., Krasinski S.D., Hirschhorn J.N., Grand R.J. (2007). Lactose and Lactase—Who Is Lactose Intolerant and Why?. J. Pediatr. Gastroenterol. Nutr..

[35] Moher D., Liberati A., Tetzlaff J., Altman D.G. (2009). Preferred Reporting Items for Systematic Reviews and Meta-Analyses: The PRISMA Statement. PLoS Med..

[36] Shamseer L., Moher D., Clarke M., Ghersi D., Liberati A., Petticrew M., Shekelle P., Stewart L.A. (2015). Preferred reporting items for systematic review and meta-analysis protocols (PRISMA-P) 2015: Elaboration and explanation. BMJ.

[37] Gil A., Fontana L., Sánchez de Medina F. (2017). Tratado de Nutrición.

[38] Montgomery R.K., Mulberg A.E., Grand R.J. (1999). Development of the human gastrointestinal tract: Twenty years of progress. Gastroenterology.

[39] Troelsen J.T. (2005). Adult-type hypolactasia and regulation of lactase expression. Biochim. Biophys. Acta Gen. Subj..

[40] Van Beers E.H., Büller H.A., Grand R.J., Einerhand A.W.C., Dekker J. (1995). Intestinal Brush Border Glycohydrolases: Structure, Function, and Development. Crit. Rev. Biochem. Mol. Biol..

[41] Newcomer A.D., McGill D.B. (1966). Distribution of Disaccharidase Activity in the Small Bowel of Normal and Lactase-Deficient Subjects. Gastroenterology.

[42] Wang Y., Harvey C., Rousset M., Swallow D.M. (1994). Expression of Human Intestinal mRNA Transcripts during Development: Analysis by a Semiquantitative RNA Polymerase Chain Reaction Method. Pediatr. Res..

[43] Boll W., Wagner P., Mantei N. (1991). Structure of the chromosomal gene and cDNAs coding for lactase-phlorizin hydrolase in humans with adult-type hypolactasia or persistence of lactase. Am. J. Hum. Genet..

[44] Naim H.Y., Lentze M.J. (1992). Impact of O-glycosylation on the function of human intestinal lactase-phlorizin hydrolase. Characterization of glycoforms varying in enzyme activity and localization of O-glycoside addition. J. Biol. Chem..

[45] Jacob R., Radebach I., Wuthrich M., Grunberg J., Sterchi E.E., Naim H.Y. (1996). Maturation of Human Intestinal Lactase-Phlorizin Hydrolase. Generation of the Brush Border form of the Enzyme Involves at Least Two Proteolytic Cleavage Steps. Eur. J. Biochem..

[46] Krasinski S., Upchurch B., Irons S., June R., Mishra K., Grand R., Verhave M. (1997). Rat lactase-phlorizin hydrolase/human growth hormone transgene is expressed on small intestinal villi in transgenic mice. Gastroenterology.

[47] Lee S.Y., Wang Z., Lin C.-K., Contag C.H., Olds L.C., Cooper A.D., Sibley E. (2002). Regulation of Intestine-specific Spatiotemporal Expression by the Rat Lactase Promoter. J. Biol. Chem..

[48] Troelsen J.T., Mehlum A., Olsen J., Spodsberg N., Hansen G.H., Prydz H., Norén O., Sjöström H. (1994). 1 kb of the lactase-phlorizin hydrolase promoter directs post-weaning decline and small intestinal-specific expression in transgenic mice. FEBS Lett..

[49] Boudreau F., Rings E.H.H.M., van Wering H.M., Kim R.K., Swain G.P., Krasinski S.D., Moffett J., Grand R.J., Suh E.R., Traber P.G. (2002). Hepatocyte Nuclear Factor-1α, GATA-4, and Caudal Related Homeodomain Protein Cdx2 Interact Functionally to Modulate Intestinal Gene Transcription. J. Biol. Chem..

[50] Krasinski S.D., Van Wering H.M., Tannemaat M.R., Grand R.J. (2001). Differential activation of intestinal gene promoters: Functional interactions between GATA-5 and HNF-1α. Am. J. Physiol. Liver Physiol..

[51] Van Wering H.M., Bosse T., Musters A., De Jong E., De Jong N., Hogen Esch C.E., Boudreau F., Swain G.P., Dowling L.N., Montgomery R.K. (2004). Complex regulation of the lactase-phlorizin hydrolase promoter by GATA-4. Am. J. Physiol. Gastrointest. Liver Physiol..

[52] van Wering H.M., Huibregtse I.L., van der Zwan S.M., de Bie M.S., Dowling L.N., Boudreau F., Rings E.H.H.M., Grand R.J., Krasinski S.D. (2002). Physical Interaction between GATA-5 and Hepatocyte Nuclear Factor-1α Results in Synergistic Activation of the Human Lactase-Phlorizin Hydrolase Promoter. J. Biol. Chem..

[53] Mitchelmore C., Troelsen J.T., Sjöström H., Norén O. (1998). The HOXC11 Homeodomain Protein Interacts with the Lactase-Phlorizin Hydrolase Promoter and Stimulates HNF1α-dependent Transcription. J. Biol. Chem..

[54] Mitchelmore C., Troelsen J.T., Spodsberg N., Sjöström H., Norén O. (2000). Interaction between the homeodomain proteins Cdx2 and HNF1alpha mediates expression of the lactase-phlorizin hydrolase gene. Biochem. J..

[55] Spodsberg N., Troelsen J.T., Carlsson P., Enerbäck S., Sjöström H., Norén O. (1999). Transcriptional regulation of pig lactase-phlorizin hydrolase: Involvement of HNF-1 and FREACs. Gastroenterology.

[56] Verhave M., Krasinski S.D., Christian S.I., Van Schaik S., van Den Brink G.R., Doting E.M.H., Maas S.M., Wolthers K.C., Grand R.J., Montgomery R.K. (2004). Regulatory Regions in the Rat Lactase-Phlorizin Hydrolase Gene that Control Cell-Specific Expression. J. Pediatr. Gastroenterol. Nutr..

[57] Wang Z., Fang R., Olds L.C., Sibley E. (2004). Transcriptional regulation of the lactase-phlorizin hydrolase promoter by PDX-1. Am. J. Physiol. Liver Physiol..

[58] Harvey C.B., Wang Y., Darmoul D., Phillips A., Mantei N., Swallow D.M. (1996). Characterisation of a human homologue of a yeast cell division cycle gene, MCM6, located adjacent to the 5′ end of the lactase gene on chromosome 2q21. FEBS Lett..

[59] Ingram C.J.E., Mulcare C.A., Itan Y., Thomas M.G., Swallow D.M. (2009). Lactose digestion and the evolutionary genetics of lactase persistence. Hum. Genet..

[60] Diekmann L., Pfeiffer K., Naim H.Y. (2015). Congenital lactose intolerance is triggered by severe mutations on both alleles of the lactase gene. BMC Gastroenterol..

[61] Szilagyi A. (2015). Adult Lactose Digestion Status and Effects on Disease. Can. J. Gastroenterol. Hepatol..

[62] Ghoshal U.C., Kumar S., Misra A., Mittal B. (2013). Lactose malabsorption diagnosed by 50-g dose is inferior to assess clinical intolerance and to predict response to milk withdrawal than 25-g dose in an endemic area. J. Gastroenterol. Hepatol..

[63] Labrie V., Buske O.J., Oh E., Jeremian R., Ptak C., Gasiūnas G., Maleckas A., Petereit R., Žvirbliene A., Adamonis K. (2016). Lactase nonpersistence is directed by DNA-variation-dependent epigenetic aging. Nat. Struct. Mol. Biol..

[64] Green F.R., Greenwell P., Dickson L., Griffiths B., Noades J., Swallow D.M., Greenwell P. (1988). Expression of the ABH, Lewis, and Related Antigens on the Glycoproteins of the Human Jejunal Brush Border. Nature.

[65] Lewinsky R.H., Jensen T.G.K., Møller J., Stensballe A., Olsen J., Troelsen J.T. (2005). T −13910 DNA variant associated with lactase persistence interacts with Oct-1 and stimulates lactase promoter activity in vitro. Hum. Mol. Genet..

[66] Olds L.C. (2003). Lactase persistence DNA variant enhances lactase promoter activity in vitro: Functional role as a cis regulatory element. Hum. Mol. Genet..

[67] Di Rienzo T., D’Angelo G., D’Aversa F., Campanale M.C., Cesario V., Montalto M., Gasbarrini A., Ojetti V. (2013). Lactose intolerance: From diagnosis to correct management. Eur. Rev. Med. Pharmacol. Sci..

[68] Tormo R., Bertaccini A., Conde M., Infante D., Cura I. (2001). Methane and hydrogen exhalation in normal children and in lactose malabsorption. Early Hum. Dev..

[69] Enattah N.S., Sahi T., Savilahti E., Terwilliger J.D., Peltonen L., Järvelä I. (2002). Identification of a variant associated with adult-type hypolactasia. Nat. Genet..

[70] Jones B.L., Raga T.O., Liebert A., Zmarz P., Bekele E., Danielsen E.T., Olsen A.K., Bradman N., Troelsen J.T., Swallow D.M. (2013). Diversity of Lactase Persistence Alleles in Ethiopia: Signature of a Soft Selective Sweep. Am. J. Hum. Genet..

[71] Baffour-Awuah N.Y., Fleet S., Montgomery R.K., Baker S.S., Butler J.L., Campbell C., Tischfield S., Mitchell P.D., Allende-Richter S., Moon J.E. (2015). Functional Significance of Single Nucleotide Polymorphisms in the Lactase Gene in Diverse US Patients and Evidence for a Novel Lactase Persistence Allele at −13909 in Those of European Ancestry. J. Pediatr. Gastroenterol. Nutr..

[72] Liebert A., Jones B.L., Danielsen E.T., Olsen A.K., Swallow D.M., Troelsen J.T. (2016). In Vitro Functional Analyses of Infrequent Nucleotide Variants in the Lactase Enhancer Reveal Different Molecular Routes to Increased Lactase Promoter Activity and Lactase Persistence. Ann. Hum. Genet..

[73] Olds L.C., Ahn J.K., Sibley E. (2011). −13915*G DNA polymorphism associated with lactase persistence in Africa interacts with Oct-1. Hum. Genet..

[74] Ingram C.J.E., Raga T.O., Tarekegn A., Browning S.L., Elamin M.F., Bekele E., Thomas M.G., Weale M.E., Bradman N., Swallow D.M. (2009). Multiple Rare Variants as a Cause of a Common Phenotype: Several Different Lactase Persistence Associated Alleles in a Single Ethnic Group. J. Mol. Evol..

[75] Røed K.H., Flagstad Ø., Nieminen M., Holand Ø., Dwyer M.J., Røv N., Vilà C. (2008). Genetic analyses reveal independent domestication origins of Eurasian reindeer. Proc. R. Soc. B Biol. Sci..

[76] Ho M.W., Povey S., Swallow D. (1982). Lactase polymorphism in adult British natives: Estimating allele frequencies by enzyme assays in autopsy samples. Am. J. Hum. Genet..

[77] Raz M., Sharon Y., Yerushalmi B., Birk R. (2013). Frequency of LCT-13910C/T and LCT-22018G/A single nucleotide polymorphisms associated with adult-type hypolactasia/lactase persistence among Israelis of different ethnic groups. Gene.

[78] Babu J., Kumar S., Babu P., Prasad J.H., Ghoshal U.C. (2010). Frequency of lactose malabsorption among healthy southern and northern Indian populations by genetic analysis and lactose hydrogen breath and tolerance tests. Am. J. Clin. Nutr..

[79] Bulhões A.C., Goldani H.A.S., Oliveira F.S., Matte U.S., Mazzuca R.B., Silveira T.R. (2007). Correlation between lactose absorption and the C/T-13910 and G/A-22018 mutations of the lactase-phlorizin hydrolase (LCT) gene in adult-type hypolactasia. Braz. J. Med. Biol. Res..

[80] Torniainen S., Parker M.I., Holmberg V., Lahtela E., Dandara C., Jarvela I. (2009). Screening of variants for lactase persistence/non-persistence in populations from South Africa and Ghana. BMC Genet..

[81] Breton G., Schlebusch C.M., Lombard M., Sjödin P., Soodyall H., Jakobsson M. (2014). Lactase Persistence Alleles Reveal Partial East African Ancestry of Southern African Khoe Pastoralists. Curr. Biol..

[82] Imtiaz F., Savilahti E., Sarnesto A., Trabzuni D., Al-Kahtani K., Kagevi I., Rashed M.S., Meyer B.F., Jarvela I. (2007). The T/G 13915 variant upstream of the lactase gene (LCT) is the founder allele of lactase persistence in an urban Saudi population. J. Med. Genet..

[83] Gerbault P. (2013). The Onset of Lactase Persistence in Europe. Hum. Hered..

[84] Gerbault P., Moret C., Currat M., Sanchez-Mazas A. (2009). Impact of Selection and Demography on the Diffusion of Lactase Persistence. PLoS ONE.

[85] Allentoft M.E., Sikora M., Sjögren K.G., Rasmussen S., Rasmussen M., Stenderup J., Damgaard P.B., Schroeder H., Ahlström T., Vinner L. (2015). Population genomics of Bronze Age Eurasia. Nature.

[86] Bersaglieri T., Sabeti P.C., Patterson N., Vanderploeg T., Schaffner S.F., Drake J.A., Rhodes M., Reich D.E., Hirschhorn J.N. (2004). Genetic signatures of strong recent positive selection at the lactase gene. Am. J. Hum. Genet..

[87] Itan Y., Powell A., Beaumont M.A., Burger J., Thomas M.G. (2009). The Origins of Lactase Persistence in Europe. PLoS Comput. Biol..

[88] Mathieson I., Lazaridis I., Rohland N., Mallick S., Patterson N., Roodenberg S.A., Harney E., Stewardson K., Fernandes D., Novak M. (2015). Genome-wide patterns of selection in 230 ancient Eurasians. Nature.

[89] Schlebusch C.M., Sjödin P., Skoglund P., Jakobsson M. (2013). Stronger signal of recent selection for lactase persistence in Maasai than in Europeans. Eur. J. Hum. Genet..

[90] Plantinga T.S., Alonso S., Izagirre N., Hervella M., Fregel R., van der Meer J.W., Netea M.G., de la Rúa C. (2012). Low prevalence of lactase persistence in Neolithic South-West Europe. Eur. J. Hum. Genet..

[91] Mathieson I., Lazaridis I., Rohland N., Mallick S., Patterson N., Alpaslan Roodenberg S., Harney E., Stewardson K., Fernandes D., Novak M. (2015). Eight thousand years of natural selection in Europe. bioRxiv.

[92] Witas H.W., Płoszaj T., Jędrychowska-Dańska K., Witas P.J., Masłowska A., Jerszyńska B., Kozłowski T., Osipowicz G. (2015). Hunting for the LCT-13910*T Allele between the Middle Neolithic and the Middle Ages Suggests Its Absence in Dairying LBK People Entering the Kuyavia Region in the 8th Millennium BP. PLoS ONE.

[93] Sabeti P.C., Schaffner S.F., Fry B., Lohmueller J., Varilly P., Shamovsky O., Palma A., Mikkelsen T.S., Altshuler D., Lander E.S. (2006). Positive natural selection in the human lineage. Science.

[94] Hedrick P.W. (2011). Population genetics of malaria resistance in humans. Heredity.

[95] Wilde S., Timpson A., Kirsanow K., Kaiser E., Kayser M., Unterländer M., Hollfelder N., Potekhina I.D., Schier W., Thomas M.G. (2014). Direct evidence for positive selection of skin, hair, and eye pigmentation in Europeans during the last 5000 y. Proc. Natl. Acad. Sci. USA.

[96] Wiley A.S. (2020). Lactose intolerance. Evol. Med. Public Health.

[97] Segurel L., Guarino-Vignon P., Marchi N., Lafosse S., Laurent R., Bon C., Fabre A., Hegay T., Heyer E. (2020). Why and when was lactase persistence selected for? Insights from Central Asian herders and ancient DNA. PLoS Biol..

[98] Durham W.H. (1991). Coevolution: Genes, Culture, and Human Diversity.

[99] Ranciaro A., Campbell M.C., Hirbo J.B., Ko W.Y., Froment A., Anagnostou P., Kotze M.J., Ibrahim M., Nyambo T., Omar S.A. (2014). Genetic origins of lactase persistence and the spread of pastoralism in Africa. Am. J. Hum. Genet..

[100] Arnold J., Diop M., Kodjovi M., Rozier J. (1980). Lactose intolerance in adults in Senegal. C. R. Seances Soc. Biol. Fil..

[101] Kuchay R.A.H. (2020). New insights into the molecular basis of lactase non-persistence/persistence: A brief review. Drug Discov. Ther..

[102] Swallow D.M., Troelsen J.T. (2016). Escape from epigenetic silencing of lactase expression is triggered by a single-nucleotide change. Nat. Struct. Mol. Biol..

[103] Oh E., Jeremian R., Oh G., Groot D., Susic M., Lee K., Foy K., Laird P.W., Petronis A., Labrie V. (2017). Transcriptional heterogeneity in the lactase gene within cell-type is linked to the epigenome. Sci. Rep..

[104] Deng Y., Misselwitz B., Dai N., Fox M. (2015). Lactose intolerance in adults: Biological mechanism and dietary management. Nutrients.

[105] Leis R., de Castro M.-J., de Lamas C., Picáns R., Couce M.L. (2020). Effects of Prebiotic and Probiotic Supplementation on Lactase Deficiency and Lactose Intolerance: A Systematic Review of Controlled Trials. Nutrients.

[106] Lomer M.C.E., Parkes G.C., Sanderson J.D. (2007). Review article: Lactose intolerance in clinical practice - myths and realities. Aliment. Pharmacol. Ther..

[107] Hertzler S.R., Savaiano D.A. (1996). Colonic adaptation to daily lactose feeding in lactose maldigesters reduces lactose intolerance. Am. J. Clin. Nutr..

[108] Szilagyi A. (2015). Adaptation to Lactose in Lactase Non Persistent People: Effects on Intolerance and the Relationship between Dairy Food Consumption and Evalution of Diseases. Nutrients.

