# Supplementary material for: Genetics of Lactose Intolerance: An Updated Review and Online Interactive World Maps of Phenotype and Genotype Frequencies
Source: Nutrients. 2020 Sep 3;12(9):2689. doi: 10.3390/nu12092689 (PMC7551416; doi:10.3390/nu12092689)
Supplement: Supplementary file 1 [file nutrients-12-02689-s001.zip › supplementary/Supplementary Figure S1-conversion.doc]

**PRISMA 2009 Flow Diagram**

**Screening**

**Included**

**Eligibility**

**Identification**

Records identified through database searching
(n = 3822)

Additional records identified through other sources
(n = 79)

Records restricted to the genetics subheading

(n = 40)

Records screened
(n = 119)

Duplicated removed

(n = 2)

Records excluded according to exclusion criteria
(n = 14)

Full-text articles assessed for eligibility
(n = 103)

Studies included in qualitative synthesis
(n = 103)

Figure S1. PRISMA 2009 Flow Diagram for adopted Systematic Review Methodology
